# Physical activity and cardiovascular risk factors in a 40- to 42-year-old rural Norwegian population from 1975–2010: repeated cross-sectional surveys

**DOI:** 10.1186/1471-2458-14-569

**Published:** 2014-06-07

**Authors:** Ane Kristiansen Solbraa, Ingar Morten Holme, Sidsel Graff-Iversen, Geir Kåre Resaland, Eivind Aadland, Sigmund Alfred Anderssen

**Affiliations:** 1 Faculty of Teacher Education and Sport, Sogn & Fjordane University College, P.O. Box 133, 6851 Sogndal, Norway; 2 Department of Sports Medicine, Norwegian School of Sport Sciences, Ullevål Stadion, P.O. Box 4014, 0806 Oslo, Norway; 3 Division of Epidemiology, Norwegian Institute of Public Health, Nydalen, P.O. Box 4404, 0403 Oslo, Norway; 4 Institute of Community Medicine, UiT The Artic University of Norway, Tromsø, Norway; 5 Faculty of Health Studies, Sogn & Fjordane University College, P.O. Box 523, 6803 Førde, Norway

**Keywords:** Physical activity, Cardiovascular disease, Risk factors, Trends, Geographic variation

## Abstract

**Background:**

Geographical differences in cardiovascular diseases (CVD) have been observed among Norwegian counties. Better long-term health status and higher physical activity (PA) levels have been documented in the county of Sogn & Fjordane compared with other counties. However, recent trends in CVD risk factors have not been documented. The aim of this study was to investigate the secular trends in leisure time physical activity (LTPA) and other CVD risk factors over a 35-year period in a rural population of 40- to 42-year-olds in western Norway and to compare these trends with national trends.

**Methods:**

Data from eight cross-sectional studies from 1975–2010 (n = 375,682) were obtained from questionnaires and physical examinations and were analyzed using mixed model regression analyses.

**Results:**

Decreasing trends were observed for sedentary behavior (for women), moderate PA, smoking, systolic blood pressure (SBP), diastolic blood pressure (DBP), high-density lipoprotein (HDL-c) and total cholesterol (TC), whereas increasing trends were observed for body mass index (BMI), triglycerides (TG), light PA, vigorous PA and sedentary behavior for men. Compared to the national trends, the trends in the 40-42-year-olds from Sogn & Fjordane were more beneficial in terms of TG, HDL-c and BMI but less beneficial in terms of SBP and DBP.

**Conclusions:**

Over a 35-year-period, this study indicates that the LTPA level has been relatively stable in the county of Sogn & Fjordane. Upward trends were observed in light and vigorous PA, whereas a downward trend was observed in moderate PA. For sedentary behavior, an upward trend was observed in men, whereas a downward trend was observed in women. For smoking, BP and cholesterol decreasing trends were found, but increasing trends were observed in BMI and TG. Compared with the national data, the trends in Sogn & Fjordane were more beneficial for TG, HDL-c and BMI but less beneficial for BP.

## Background

In the 1970s, Norway had a high cardiovascular disease (CVD) mortality rate compared to the present rate, even in an international context [1]. Considerable variation in the CVD mortality rates among Norwegian counties have been observed [1], and the county of Sogn & Fjordane had the lowest CVD mortality rate in Norway from 1964–1975 [1].

As of 2013, Sogn & Fjordane has a population of approximately 110,000 inhabitants, and people mainly live in small urban areas or are scattered over a wide area [2]. Furthermore, the divorce rate is lower [3], the education level is higher, and the unemployment rate is lower compared with the rest of Norway [4]. The life expectancy is currently 78.3 years for men and 83.6 years for women, compared with the national averages of 77.2 years for men and 82.2 years for women [4].

Beginning in 1975, the National Health Screening Service (NHSS) implemented a screening program for CVD risk. The results from this program have shown that Sogn & Fjordane county had one of the lowest levels of infarct risk in Norway until 1995 [5].

Inconsistent results have been found in studies investigating secular trends in leisure time physical activity (LTPA) [6-9]. A substantial decrease in the prevalence of smoking has been found in both sexes [6,10]. An increase in body mass index (BMI) has been observed in all segments of the population during recent decades, both in Norway [6,10-12] and in most other Western countries [13]. Furthermore, blood pressure (BP) and total cholesterol (TC) levels have decreased in Norway [10] and in the majority of other Western countries [14,15].

Understanding the geographic variation in CVD risk factors is important because it may provide useful information about implementing interventions to improve cardiovascular health at the community level. Because of the positive long-term health status of the population of Sogn & Fjordane and the shift in traditional CVD risk factors, it is interesting to investigate whether this region has maintained a better risk factor status than the rest of Norway. Thus, the aim of this study was to investigate the secular trends in LTPA and other CVD risk factors over a 35-year period in a 40- to 42-year-old healthy rural population in western Norway and to compare these trends with national trends over the same time period.

## Methods

### Population

Data from Sogn & Fjordane were collected in seven cross-sectional studies by the NHSS (1975–1999) and The Physical Activity among Adults and Older People Study (2010), a multicenter study conducted by the Norwegian School of Sport Sciences. All eight studies invited all 40- to 42-year-olds to participate (for the last cohort in three municipalities, whereas a randomly selected sample of 40-42-year-olds were invited throughout Norway). The dataset (n = 21,372) includes data from all 26 municipalities in Sogn & Fjordane (Table 1). The response rate varied from 92% in 1975 to 60% in 2010. To compare Sogn & Fjordane with the rest of the country, data from the other 18 counties in Norway (n = 354,310) were used. The participation rate ranged from 32% to 94%, with a decreasing trend from 1975 to the present [5,16]. The population details have been described elsewhere [5,17].

**Table 1:** **Overview of the study population in Sogn & Fjordane**, **n**, **participation rate** (%)

**Cohort**, **year**	**Invited**	**Attended**	**Participation rate (%)**
Cohort I, 1975	2781	2564	92
Cohort II, 1980	2940	2571	87
Cohort III, 1985	3796	3060	81
Cohort IV, 1990	3904	3228	83
Cohort V, 1993	4338	3261	75
Cohort VI, 1996	4402	3264	74
Cohort VII, 1999	4433	3091	70
Cohort VIII, 2010	553	333	60
In total	27147	21372	79

In the cohorts from 1975–1999, the data were gathered using questionnaires and physical examinations. The participants were invited to a health screening, and a survey questionnaire collected self-reported LTPA, smoking and education level. The examinations included weight, height and BP measurements and blood sample collections. The surveys were administered by the NHSS mobile teams [5].

In the cohort in 2010, the data were collected by postal mail for all participants, whereas a physical examination was conducted on random selected 1/3 of the participants. A questionnaire covered self-reported LTPA, smoking, weight, height and educational level. Two experienced nurses measured weight, height and BP and collected blood samples [17].

The participants provided written informed consent [17]. The Norwegian Institute of Public Health has given their approval to use the data from the National Health Screening Service. The study was approved by the Regional Committee for Medical Research Ethics, the Norwegian Social Science Data Services AS and the Norwegian Tax Department.

### Measures

#### Physical activity

In all cohorts, except in 1996, LTPA was self-reported using a questionnaire developed in Gothenburg [18]: *Please note your exercise and physical exertion in leisure time. If the activity varies considerably*, *e.g. between summer and winter*, *then give an average. The question applies to the past year. Tick* “*YES*” *beside the description that fits best*: (*1*) *Reading*, *watching TV or other sedentary activity*?; (*2*) *Walking*, *cycling*, *or other forms of exercise at least 4 hours per week*? (*Including walking or cycling to place of work*, *Sunday*-*walking*, *etc*.); (*3*) *Participation in recreational sports*, *heavy gardening*, *etc*.? (*Note*: *duration of activity at least 4 hours a week*); (*4*) *Participation in hard exercise or sports competitions*, *regularly several times a week*? [18-20]. LTPA was then classified in four categories: 1) sedentary behavior, 2) light physical activity (PA), 3) moderate PA and 4) vigorous PA.

#### Cardiovascular disease risk factors

In the cohorts from 1975–1999, weight and height were measured to the nearest 0.5 kg and 1 cm [21,22]. In the 2010 cohort, weight and height were both measured to the nearest 0.1 kg and 0.5 cm (n = 115) and self-reported (n = 333) [17]. Body composition was expressed as BMI (kg/m^2^).

Systolic BP (SBP) and diastolic BP (DBP) were measured manually using the mercury method (Erkameter, ERKA, Kallmeyer Medizintechnik GmbH & Co.KG, Bad Tölz, Germany) in 1975 and 1980. Measurements were taken two times with a one-minute interval between the measurements [23]. In the 1985–1999 cohorts, the measurements were taken automatically using a Dinamap BP monitor (Critikon Cooperation, Tampa, Florida, USA) [24]. In the 2010 cohort, BP was measured automatically using an Omron HEM-907 BP monitor (Omron Healthcare, Inc., Vernon Hills, IL, US) in Sogn & Fjordane [17] and a manual sphygmomanometer (Big Ben, Reister, Junginen, Germany) in the other counties [25]. From 1985 onward, three repeated measurements were taken at one-minute intervals for all BP measurements [17,24]. The mean of the two last measurements was used in the statistical analyses. The validations of the Erkameter and the Dinamap have been described elsewhere [23]. Tests of agreement between the Omron and; Dinamap and Big Ben respectively showed small differences.

Non-fasting intravenous blood samples were taken from the antecubital vein. The samples were centrifuged immediately and sent to the Department of Medical Biochemistry at Oslo University Hospital, Ullevaal (Norway), where they were analyzed for TC and triglycerides (TG) (all cohorts) and high-density lipoprotein (HDL-c) (in 1980–2010) [5,17]. In 2010, blood samples were only taken in the county of Sogn & Fjordane [17]. Smoking was self-reported in all cohorts. To estimate the total 10-year risk of CVD mortality, the NORRISK model was used. The NORRISK model is based on Norwegian mortality data and risk profiles and contains of the following five risk factors: sex, age, TC, SBP and smoking [26]. Education data were dichotomously scored as 1) high school or less or 2) college or university.

### Statistics

The descriptive results are presented as means and 95% confidence intervals (CI) (continuous data) or proportions (%) (categorical data). To investigate trends over time in the county of Sogn & Fjordane, we used two-level linear (continuous data) and logistic (categorical data) mixed model regression analyses with random intercepts for municipalities (n = 26) and with time (actual year) as the fixed variable. Because a non-linear trend was found for smoking, a categorical time variable (seven time-points) was used. The effect of sex was tested by including the main effect and the interaction effect (sex*time) in the models. Because only three municipalities were represented in 2010, mixed models were employed to detect any differences in the trends between the three municipalities and the rest of the county from 1975–1999. Due to the substantial differences in the population size, a trend analysis was performed from 1975–1999, and the developments from 1999–2010 were described by analyzing the levels of risk factors. To compare the trends in the county of Sogn & Fjordane with those in the rest of Norway, we applied two-level mixed model regression analyses including random intercepts for the counties (n = 19) (because a municipality variable was unavailable in the rest of Norway) and fixed effects for the county of Sogn & Fjordane vs. the rest of Norway. Because the non-fasting TG data were skewed, log-transformed data were used in the analysis, and the LnTG was adjusted for the amount of time since the last meal, with three hours as the reference. Bland-Altman plots and intra-class correlation coefficients were used to investigate the agreement between measured and self-reported weight and height in 2010 and to investigate the agreement between the two automatic BP instruments (Dinamap and Omron) and the two BP instruments used in 2010 (Omron and Big Ben). The BMI values were adjusted for the measurement method and for smoking, but the crude results are presented because the differences were minor. The BP data were corrected for the BP measurement instrument with the Omron as the reference. The results are reported as changes (continuous variables) and odds ratios (OR, categorical variables) with 95% CIs and observed significance levels. The analyses were performed using STATA version 12 (StataCorp, College Station, TX, USA).

## Results

### Trends in Sogn & Fjordane

**Figure 1:**
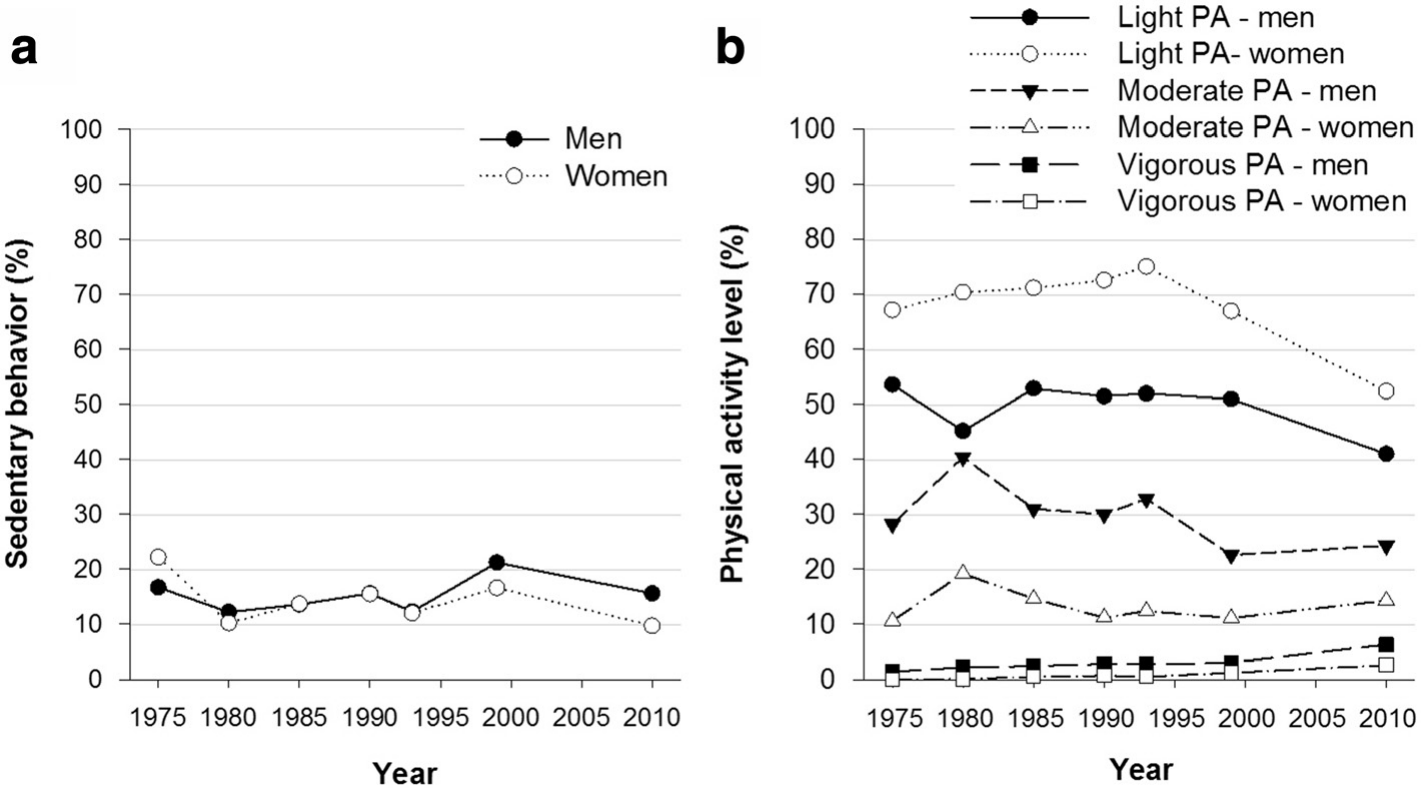
**Trends in leisure time physical activity in Sogn & Fjordane 1975–2010. (a)** Sedentary behavior and **(b)** physical activity, stratified by sex, proportion (%).

**Figure 2:**
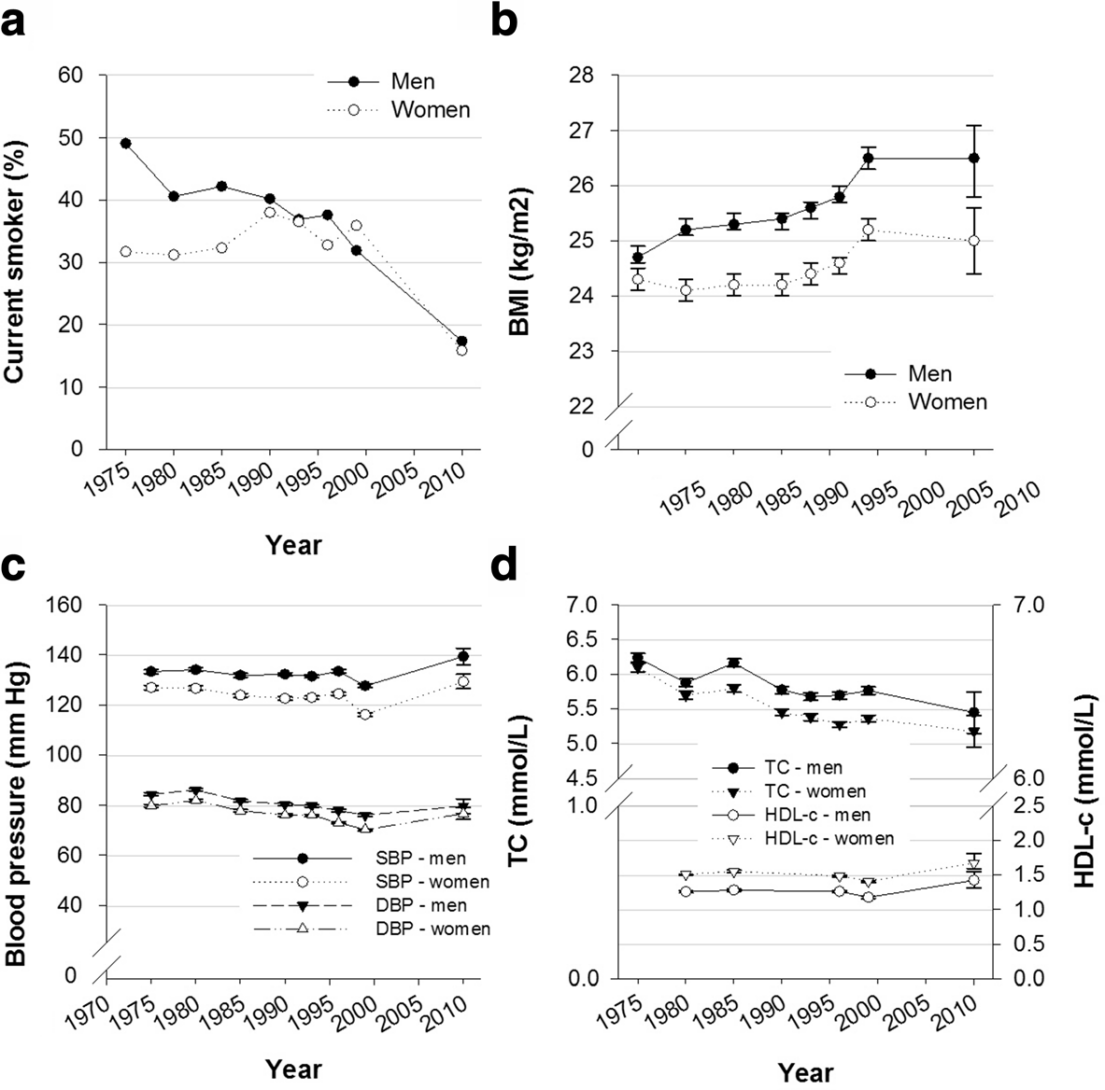
**Trends in other risk factors in Sogn & Fjordane 1975**–**2010. (a)** Smokers, **(b)** BMI, **(c)** blood pressure and **(d)** TC and HDL-c, stratified by sex, proportion (%) or mean (95% CI). Body mass index; BMI, systolic blood pressure; SBP, diastolic blood pressure; DBP, total cholesterol; TC and high density lipoprotein; HDL-c.

Table 2 presents the proportion of men and women who reported sedentary, light, moderate and vigorous PA in each cohort. In total, LTPA has been relatively stable over 35 years. For sedentary behavior, no significant trend was found from 1975–1999 for men and women combined (Table 3). However split by sex, a significant decreasing trend was observed for women (OR 0.992, 95% CI: 0.985,0.999), whereas an increasing trend was found for men (OR 1.012, 95% CI: 1.004,1.020). For both sexes, a decrease was observed from 1999–2010 (Figure 1a). A significant increasing trend was observed in light (for women only) and vigorous PA (for both sexes) from 1975–1999 (Table 3). From 1999–2010, there was a decrease in light PA and a further increase in vigorous PA (Figure 1b). A significant decreasing trend in moderate PA was observed for both sexes from 1975–1999 (Table 3), however, this trend increased to 2010 (Figure 1b). A significant decreasing trend in smoking was observed among men from 1975–1999, whereas a significant increasing trend was observed among women (Figure 2a). Thereafter, there was a decrease in smoking for both sexes. Over 35 years smoking decreased by 31.7% for men and 15.8% for women. The mean BMI (Figure 2b) increased between 1975–1999 for both sexes, but the trend leveled off thereafter. Over 35 years BMI has increased by 1.8 kg/m^2^ for men and 0.7 kg/m^2^ for women. 55.6% men and 44.4% women were overweight or obese in 2010 compared to 40.9% and 34.5% in 1975. With regard to SBP and DBP, a significant decreasing trend was observed for both sexes from 1975–1999 (Figure 2c). From 1999–2010, an increase was observed for both sexes (corresponding to 12.1 mmHg for SBP and 5.1 mmHg for DBP). Table 2 presents the proportion of men and women who were smokers, were overweight or obese or had hypertension in each cohort.

**Table 2:** **Descriptive data on physical activity level and other risk factors in Sogn & Fjordane**, **stratified by sex**, %

		**1975**	**1980**	**1985**	**1990**	**1993**	**1996**	**1999**	**2010**
**Sedentary behavior**	**Men**	16.7	12.2	13.6	15.6	12.3	---	21.2	15.6
	**Women**	22.1	10.2	13.7	15.5	12.0	---	16.6	9.7
**Light PA**	**Men**	53.6	45.2	53.0	51.6	52.0	---	52.5	48.4
	**Women**	67.2	70.5	71.3	72.6	75.1	---	70.4	68.3
**Moderate PA**	**Men**	28.2	40.4	31.0	30.0	32.8	---	23.2	27.7
	**Women**	10.6	19.2	14.6	11.2	12.4	---	11.7	18.6
**High PA**	**Men**	1.4	2.2	2.4	2.8	2.8	---	3.1	7.4
	**Women**	0	0.1	0.5	0.7	0.5	---	1.3	3.4
**Smokers**	**Men**	49.1	40.6	40.2	40.2	36.9	37.6	31.9	17.4
	**Women**	31.7	31.2	32.3	38.0	36.5	32.8	35.9	15.9
**BMI ≥25 kg/m**^**2**^	**Men**	40.9	46.5	51.0	51.3	53.5	58.7	64.7	55.6
	**Women**	34.5	30.0	33.0	32.5	35.8	34.8	43.3	44.4
**SBP >140 mmHg**	**Men**	26.8	28.6	22.8	22.8	19.8	24.7	16.7	38.3
	**Women**	17.1	16.0	13.0	9.7	10.3	10.1	5.6	16.7
**TC >5.0 mmol/L**	**Men**	86.5	79.9	85.8	76.2	73.0	74.3	76.1	63.6
	**Women**	85.2	75.3	79.4	65.3	62.5	58.8	61.5	48.4
**HDL-c ≤0.90 mmol/L (men), ≤1.00 mmol/L (women)**	**Men**	---	10.4	6.8	---	---	9.6	15.6	9.1
	**Women**	---	4.2	2.1	---	---	5.2	10.0	6.5
**TG > 1.7 mmol/L**	**Men**	46.6	43.1	48.8	47.9	49.6	47.4	50.2	38.6
	**Women**	15.7	12.4	16.8	19.1	18.5	16.9	20.6	9.7

*PA*, physical activity; *BMI*, body mass index; *SBP*, systolic blood pressure; *TC*, total cholesterol; *HDL*-c, high density lipoprotein; *TG*, triglyceride.

**Table 3:** **Trend in levels of physical activity in Sogn & Fjordane from 1975**-**1999**

	**Change**	**95% CI**	**p**-**value**
Gothenburg instrument LTPA			
Sedentary behavior	1.002	(0.997, 1.007)	0.481
Light PA	1.007	(1.003, 1.011)	≤0.001
Moderate PA	0.985	(0.981, 0.990)	≤0.001
High PA	1.037	(1.021, 1.054)	≤0.001

95% *CI*, 95% confidence interval; *LTPA*, leisure time physical activity; *PA*, physical activity. All data are yearly average ORs.

Significant decreasing trends in TC and HDL-c were observed for both men and women between 1975 and 1999 (Figure 2d). From 1999–2010, an additional decrease in TC was observed, whereas an increase in HDL-c was observed for both sexes. Over 35 years TC decreased by 0.79 mmol/L for men and 0.92 mmol/L for women, whereas HDL-c increased by 0.16 mmol/L for both sexes from 1980–2010. A significant increasing trend in TG was observed between 1975 and 1999, but TG decreased for both sexes after 1999. College or university education in Sogn & Fjordane increased from 26% in 1996 to 37% in 2010. Table 2 presents the proportion of men and women who had TC, HDL-c and TG levels above clinical cut-offs in each cohort.

### Trends in sogn & fjordane compared with national trends

**Figure 3:**
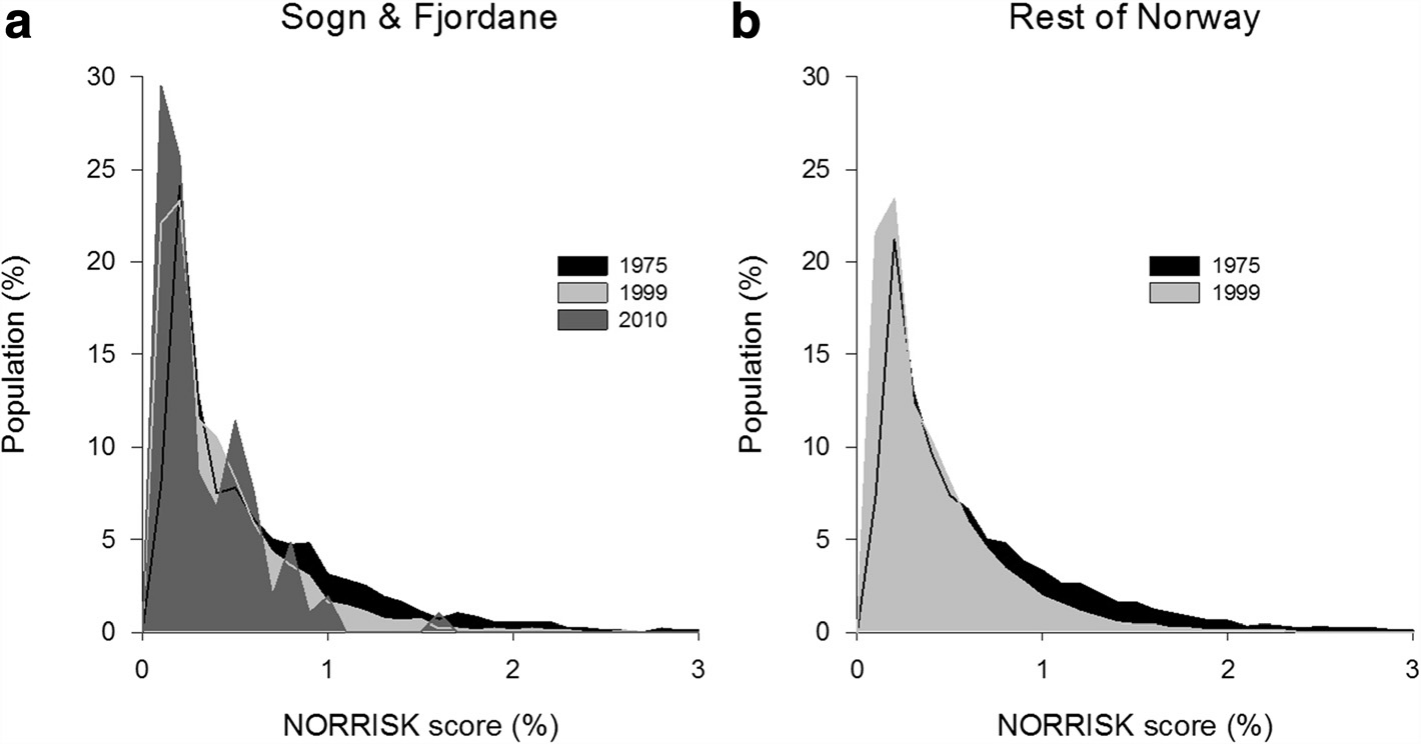
**The NORRISK score. **The distribution of total CVD risk (%) in **(a)** Sogn & Fjordane 1975-2010 and **(b)** the rest of Norway 1975-1999.

Sogn & Fjordane had more beneficial trends for TG, HDL-c and BMI but less beneficial trends for SBP and DBP compared with the rest of Norway (Table 4). No difference in trend was found for PA between Sogn & Fjordane and the rest of Norway. . The distribution of the total 10-year risk of CVD mortality in Sogn & Fjordane (1975, 1999 and 2010) and in the rest of Norway (1975 and 1999), as estimated by the NORRISK score, is presented in Figure 3a-b. The decrease in the total CVD risk was marginally but significantly greater in the rest of Norway than in Sogn & Fjordane from 1975–1999 (Table 4). This difference corresponds to a 0.085% decrease in the total risk of CVD mortality nationally compared with a 0.069% decrease in Sogn & Fjordane over 10 years.

**Table 4:** **Trend in risk factors in Sogn & Fjordane versus the rest of Norway from 1975**-**1999**

	**Trend Sogn & Fjordane**			**Trend rest of Norway**			**Difference in trend**		
	**Change**	**95% CI**	**p**-**value**	**Change**	**95% CI**	**p**-**value**		**95% CI**	**p**-**value**
BMI (kg/m^2^)	0.04	(0.04, 0.05)	≤0.001	0.08	(0.08, 0.08)	≤0.001	−0.04	(−0.03, −0.04)	≤0.001
SBP (mmHg)*	−0.26	(−0.29, −0.24)	≤0.001	−0.32	(−0.34, −0.31)	≤0.001	0.06	(0.09, 0.04)	≤0.001
DBP (mmHg)*	−0.41	(−0.43, −0.40)	≤0.001	−0.45	(−0.46, −0.45)	≤0.001	0.04	(0.06, 0.02)	≤0.001
HDL-c (mmol/L)	−0.003	(−0.004, −0.002)	≤0.001	−0.004	(−0.005, −0.004)	≤0.001	0.001	(0.002, 0.000)	0.045
TG (mmol/L)**	0.002	(0.001, 0.003)	0.002	0.004	(0.004, 0.005)	≤0.001	−0.003	(−0.002, −0.004)	≤0.001

95% *CI*, 95% confidence interval; *BMI*, body mass index; *SBP*, systolic blood pressure; *DBP*, diastolic blood pressure; *HDL*-c, high density lipoprotein; *TG*, triglyceride. All data are yearly average changes. *SBP and *DBP* corrected for *BP* device with Omron as reference instrument, **TG adjusted for time since last meal with three hours as a reference.

## Discussion

Overall, the LTPA level in Sogn & Fjordane has been relatively stable over a 35-year period. A beneficial trend was observed in terms of sedentary behavior (for women), light and vigorous PA, but commitment to moderate PA decreased and sedentary behavior increased (among men). With respect to smoking, BP and cholesterol decreasing trends were found, but increasing trends were observed in BMI and TG. Although some of the changes seems minor, changes are large in the population as a whole [27]. Compared with the national data, the trends in Sogn & Fjordane were more beneficial for TG, HDL-c and BMI but less beneficial for BP.

Trend data on PA are scarce [9], and inconsistent results have been found for LTPA in previous studies [6-9]. The observed LTPA trend may indicate a shift from recreational walking toward specific training activities. Our study partly corroborate previous observations by Anderssen et al. who also found a downward trend for sedentary behavior (for women) and moderate PA (for men) and an upward trend for sedentary behavior (for men) and vigorous PA [6], Borodulin et al. [7] found an increasing trend in LTPA during the same period. Both these studies used the same PA questionnaire as in our study. The population in Sogn & Fjordane have reported lower levels of sedentary behavior and higher levels of physical activity compared to the rest of Norway for decades [5,17,28]. However, no differences in trend between the areas were observed. The increasing trend in sedentary behavior for men and the overall decreasing trend in moderate PA in Sogn & Fjordane might signal a transition toward less physically demanding work in addition to a motorized transportation pattern. The observed decrease in smoking confirms previous studies [6,10], and the pattern in Sogn & Fjordane over the last 35 years is similar to that in the rest of Norway.

The steady increase in BMI for men and the slightly U-shaped trend for women confirm the BMI trends identified in other national studies [5,6,10,11]. A substantial acceleration in the BMI increase was observed in the beginning of the 1990s for both sexes, and may be associated with the acceleration of the digital revolution happening at the same time. The decrease in smoking prevalence could also partly explain the BMI increase. Anderssen et al. [6] found that the increase in BMI was significantly larger in sedentary individuals compared with those who were more physically active. The development is explained as most likely caused by a changing environment that promotes calorie intake and counteracts energy expenditure [6]. Recent studies have indicated a possible slowing of the obesity epidemic [12,29]. Our study indicates that BMI values have plateaued during the last decade. Midthjell et al. [12] did not find a plateau in BMI in another Norwegian population, but they did observe a plateau in the proportion of the population that was overweight. The leveling off observed in BMI, might be related to the observed decrease in sedentary behavior and increace in moderate and vigorous PA from 1999–2010. The decreasing trend found in SBP and DBP in both Sogn & Fjordane and the rest of Norway is supported by national [10] and international [14] studies. The less beneficial trend in BP in Sogn & Fjordane compared with the rest of Norway might be the result of a delayed development in an already healthy population. In Norway, the substantial decrease in BP in the late 1990s has previously been discussed as a possible methodological issue, at least in part [24]. The use of antihypertensive medications has increased from 2004–2010 [30], but in 2010 there was no difference between Sogn & Fjordane and the rest of the country (data not shown).

There is no significant difference in the TC trend between Sogn & Fjordane and the rest of Norway. With regard to HDL-c however, the trend in Sogn & Fjordane is slightly more beneficial, which may be explained by the more beneficial LTPA. Lipid data from the 2008 HUNT study [31] revealed that TC levels were similar for men but lower for women in Sogn & Fjordane than in central Norway and that HDL-c levels were higher for both men and women in Sogn & Fjordane. The higher BMI in central Norway could be a possible explanation. However, the possible overrepresentation of a physically active population in the 2010 cohort could explain both the lower BMI and the more beneficial lipid levels in Sogn & Fjordane compared with the HUNT population.

The nationwide decrease in total CVD risk expressed by the NORRISK score indicates that the CVD risk profile in Norway has improved over time. The less beneficial trend in Sogn & Fjordane, however, may indicate that the healthy population is becoming more similar to the rest of Norway. In addition, the differences between counties in total and CVD mortality have decreased in recent decades [4].

### Strengths and limitations

The long time span (including eight cohorts), the large populations with high participation rates in the majority of the cohorts and consistent measurement and analyses used for most variables are strengths of this study.

The study also has limitations. First, the number of participants and the participation rate in the 2010 cohort introduced uncertainty regarding the most recent period and could be a source of selection bias. Second, investigating trends over a 35-year span provides measurement challenges. Multiple BP devices have been used, but device comparisons have been performed to minimize potential bias and to correct for the instruments used. The use of self-reported weight and height for parts of the population in 2010 creates some uncertainty. However, tests of agreement between the self-reported and measured weights and heights from 2010 produced satisfactory results, and adjusting for the measurement method yielded results similar to the crude rates. In addition, using self-reported LTPA is a potential source of recall and social desirability bias [32]. Although the questions were slightly modified to adapt to one’s perception of terms, uncertainty related to changes over time in the participants’ interpretation of the term LTPA remains to some degree [6,32]. However, currently no data are available from investigations of secular trends in LTPA using objective measurements in Norwegian adults. Interaction with sex was found for BMI, SBP, DBP, TC, HDL-c, smoking, sedentary behavior and vigorous PA. Descriptive data are presented for both sexes. However, due to less evident interactions for the majority of the variables and for the readability, trend data in tables are presented in total, whereas trends for men and women are presented separately in text where appropriate.

## Conclusions

In sum, we found that the LTPA level has been relatively stable over a 35-year period, also in Sogn & Fjordane, the county that has been considered the healthiest in Norway. Upward trends were observed in light and vigorous PA, whereas a downward trend was observed in moderate PA. For sedentary behavior, an upward trend was observed in men, whereas a downward trend was observed in women. With respect to smoking, BP and cholesterol decreasing trends were found, but increasing trends were observed in BMI and TG. Compared with the national data, the trends in Sogn & Fjordane were more beneficial for TG, HDL-c and BMI but less beneficial for BP.

## Competing interests

The authors declare that they have no competing interests.

## Authors’ contributions

AKS participated in the design of the study, drafted the manuscript and performed the statistical analysis and data interpretation. IMH participated in the design of the study and contributed to the statistical analysis. SGI participated in the design of the study. GKR participated in the design of the study. EA contributed to the statistical analysis. SAA was the lead investigator and participated in the design of the study. All authors contributed to writing the manuscript, the data interpretation and read and approved the final manuscript.

## Pre-publication history

The pre-publication history for this paper can be accessed here:

http://www.biomedcentral.com/1471-2458/14/569/prepub
